# Conserved non-coding elements: developmental gene regulation meets genome organization

**DOI:** 10.1093/nar/gkx1074

**Published:** 2017-11-07

**Authors:** Dimitris Polychronopoulos, James W. D. King, Alexander J. Nash, Ge Tan, Boris Lenhard

**Affiliations:** 1 Computational Regulatory Genomics Group, MRC London Institute of Medical Sciences, Du Cane Road, London W12 0NN, UK; 2 Institute of Clinical Sciences, Faculty of Medicine, Imperial College London, Hammersmith Campus, Du Cane Road, London W12 0NN, UK; 3 Sars International Centre for Marine Molecular Biology, University of Bergen, Thormøhlensgate 55, N-5008 Bergen, Norway

## Abstract

Comparative genomics has revealed a class of non-protein-coding genomic sequences that display an extraordinary degree of conservation between two or more organisms, regularly exceeding that found within protein-coding exons. These elements, collectively referred to as conserved non-coding elements (CNEs), are non-randomly distributed across chromosomes and tend to cluster in the vicinity of genes with regulatory roles in multicellular development and differentiation. CNEs are organized into functional ensembles called genomic regulatory blocks–dense clusters of elements that collectively coordinate the expression of shared target genes, and whose span in many cases coincides with topologically associated domains. CNEs display sequence properties that set them apart from other sequences under constraint, and have recently been proposed as useful markers for the reconstruction of the evolutionary history of organisms. Disruption of several of these elements is known to contribute to diseases linked with development, and cancer. The emergence, evolutionary dynamics and functions of CNEs still remain poorly understood, and new approaches are required to enable comprehensive CNE identification and characterization. Here, we review current knowledge and identify challenges that need to be tackled to resolve the impasse in understanding extreme non-coding conservation.

## INTRODUCTION

**Figure 1.**
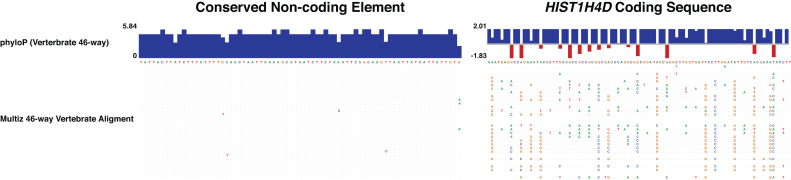
The phenomenon of extreme non-coding conservation. A conserved CNE (Human–Tetraodon CNE, on the left) shown here is more conserved than a protein-coding sequence (*HIST1H4D*, on the right). The multiple sequence alignment of 46 vertebrate species and the corresponding phyloP scores illustrate the evolutionary conservation of the CNE and protein-coding sequence. PhyloP scores range from negative to positive scores (red to blue) and indicate positive and negative selective pressure respectively. The 46-way alignment was downloaded from the UCSC genome browser and spans ∼600 million years of evolution since the last common ancestor of humans and lampreys.

Extremely conserved sequences within the non-coding portion of metazoan genomes were initially identified more than three decades ago by comparing the introns and UTRs of mammalian and avian mRNAs (1–5). These pioneering studies identified elements that had maintained >70% sequence identity over hundreds of millions of years of evolution, far greater than that expected for neutrally evolving DNA. Progress in DNA sequencing technologies aided the further identification of numerous individual examples of non-coding conservation (6–9). The prevalence of these elements was only truly appreciated when multiple groups published the systematic, genome-wide identification of conserved non-coding elements (CNEs) (10–12). This established that there are hundreds to thousands of extremely conserved non-coding elements identifiable across more than 400 million years of evolution that, in many cases, exhibit levels of conservation well beyond those seen in protein-coding genes (Figure 1).

Since then, numerous studies have defined sets of evolutionarily conserved CNEs, each using different conservation criteria, species comparisons and nomenclature (summarized in Supplementary Table S1). Our current understanding is that these overlapping sets represent the same elements, maintained by similar, poorly understood processes. Therefore, we collectively refer to those elements as CNEs. As conservation is dependent on the species compared, elements can be lineage-specific. For example, not all CNEs identified by comparing mammalian genomes appear conserved when the same conservation criteria are used on more distant genome comparisons. Additionally, all CNEs detected among closely related species (e.g. human and mouse) may not be functional elements, whereas an overwhelming majority of the CNEs conserved between distant species are likely to be functional.

A handful of resources, mainly databases, exist which contain pre-computed sets of CNEs (13–19) (Table 1). These databases contain many of the CNE sets studied so far; their disadvantage is that they are static and seldom updated.

**Table 1.** Conserved non-coding elements (CNE) resources

Abbreviation	Description	Identification	Reference
ANCORA	Atlas of non-coding conserved regions in animals	≥70% seq. id. over 30 or 50 nt in different metazoa	(13)
CEGA	Conserved elements from genomic alignments	threshold-free phylogenetic modeling	(14)
cneViewer	Conserved non-encoding element viewer	user-specified	(15)
CONDOR	COnserved Non-coDing Orthologous Regions database	multiple and multi-pairwise alignments of orthologous regions between *Fugu* and human, mouse, rat, and dog; coding and repetitive regions are removed	(16)
UCbase	Ultraconserved elements database	100% seq. id. over 200 nt between human and mouse	(17)
UCNEbase	Ultraconserved CNEs	≥95% seq. id. over 200 nt in the human and chicken genomes; coding regions are removed	(18)
VISTA	ViSualization tool for alignment	extremely conserved sequences between human and rodents that have been tested *in vivo* for enhancer activity	(19)

In this review, we provide a comprehensive account of the genomic organization of CNEs and their intriguing sequence properties. We discuss CNE functions, their roles in disease aetiology and hypotheses regarding their emergence and evolutionary dynamics. We conclude with unaddressed questions important for our progress in understanding these elements in the future.

## KNOWN FUNCTIONS OF CONSERVED NON-CODING ELEMENTS

### Many CNEs function as developmental enhancers

A pioneering study by Aparicio *et al.* published more than two decades ago identified one of the first CNEs, and at the same time demonstrated that it exhibited enhancer activity in a transgenic mouse model (20). Since then, reporter gene assays throughout the vertebrate phylogeny, from mouse (21–24), chicken (25,26), frog (27) to zebrafish (28–30) have demonstrated that CNEs typically function as enhancers in various developmental contexts. This has led to the view of CNEs as *cis*-regulatory elements coordinating spatial-temporal gene expression, especially during embryonic development (11,31,32). While the majority of CNEs act as enhancers, it should be noted that not all functional enhancers display such extreme levels of conservation as CNEs (33,34), including many enhancers found within dense genomic clusters of CNEs.

In line with CNEs predominantly being developmental enhancers, detectable phenotypic changes have been associated with alterations in CNEs. A particularly well-characterized case is the *SHH* ZRS enhancer, in which point mutations result in preaxial polydactyly in both human and mouse (35–38). Mutations in a CNE proximal to the *HMX1* gene cause aberrant external ear development in wild populations of rats and highland cattle (39). A mouse sequence called *M280*, which contains a CNE identical between human, mouse and rat, is indispensable for body growth in mice (40). Many more cases linking CNEs to both human disease and lineage-specific traits are discussed in more depth in the ‘*Diseases associated with non-coding conservation’* and ‘*CNE Modifications and Losses*’ sections. They highlight the important role of CNEs as developmental enhancers.

## GENERAL FEATURES OF NON-CODING CONSERVATION

### Genomic organization of conserved non-coding elements

**Figure 2.**
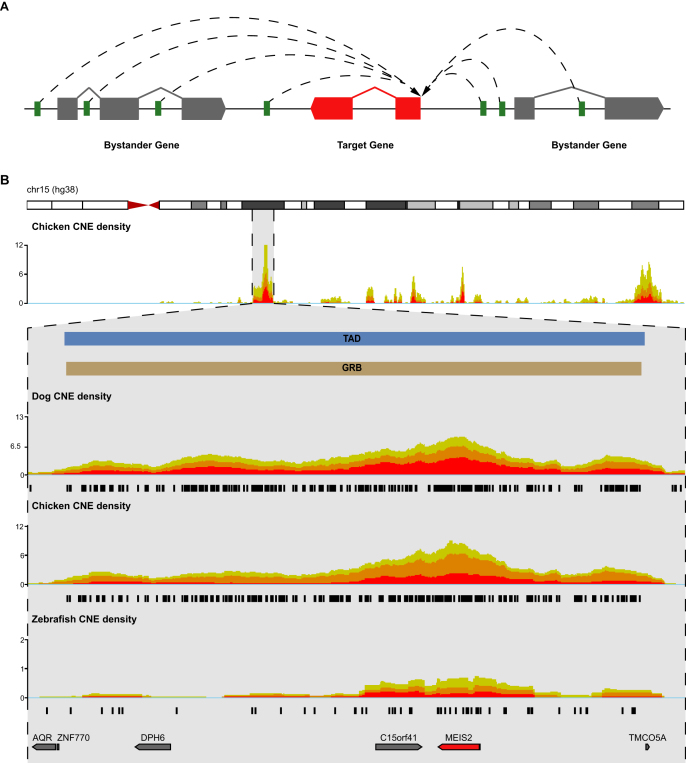
(**A**) The GRB model. The regulatory input for one or more target genes (red) is provided by long-range interactions (dashed lines) between CNEs (green) and the target gene’s promoter. Bystander genes (gray) often contain CNEs in their introns but remain unresponsive to CNE-mediated regulation. For more details, see text, (**B**) CNE clustering across chromosome 15 and at the *MEIS2* locus (shown zoomed in below the whole chromosome track). The human *MEIS2* GRB (brown) is a 3.3 Mb region defined by an array of conserved non-coding elements. *MEIS2* (red) encodes a transcription factor involved in lens development through regulation of *PAX6*. Regardless of species used in the pairwise comparison against human, CNEs (black) clearly mark the boundaries of this GRB. The boundaries of the topologically associated domain (TAD) covering MEIS2 (TAD in blue; H1-ESC TAD calls are generated using *HMM_calls*) and the GRB spanning this locus are highly concordant.

One of the most striking features of CNEs is their non-random distribution across genomes (10,11,41–43). CNEs reside in clusters (12) that often span regions with low gene density, including gene deserts (44). These clusters tend to coincide with key developmental regulatory *target* genes, such as *SHH* and *HMX1* mentioned above, with CNEs driving the expression of these target genes without affecting unrelated *bystander* genes within the cluster (11,30,45,46). CNEs, their target genes, and associated bystanders are maintained in syntenic blocks due to the requirement for regulatory elements to remain in *cis* with their target genes. This has constrained the evolution of metazoan genomes, resulting in arrays of syntenic CNEs that form functional, long-range, gene regulatory modules. The regions spanned by these arrays are named *genomic regulatory blocks* (GRBs) and, in addition to the array of CNEs, always contain a target gene regulated by the CNEs. Some, but not all, GRBs also contain bystander genes. Bystander genes frequently contain CNEs within their introns; however, they are unresponsive to CNE regulation due to differences in their promoter architecture (45,46). GRB target genes share defining properties that distinguish them from bystander genes. In vertebrate genomes, these include: (i) longer CpG islands, often several of them bound by Polycomb proteins, (ii) distinct patterns of histone modifications, (iii) differences in the distribution of alternative transcription start sites and (iv) a characteristic spatial organization of transcription factor binding sites (TFBS) in their core promoters (46). In *Drosophila*, GRB target genes are also characterized by broad Polycomb binding, and longer than average introns that often contain CNEs (45). The GRB model is depicted schematically in Figure 2A. The *MEIS2* target gene is presented in Figure 2B as part of a GRB with a well-characterized regulatory landscape.

#### Relationship with TADs

Recent studies in bilaterian genomes have led to the identification of genomic regions within which frequent chromatin interactions occur. These regions, known as TADs, form distinct genomic boundaries within which preferentially self-interacting regions are enriched (47–49). TAD boundaries are largely invariant across cell types (48,50,51) and between species (52). This robustness and prevalence of TADs prompted Harmston *et al.* to investigate whether TADs and GRBs reflect the same underlying phenomenon (53); see also Figure 2B. They demonstrated that GRB boundaries are resilient to CNE identification thresholds and the evolutionary distance of the species comparison used (53). Further, GRB boundaries coincide with TAD boundaries around developmental genes in both vertebrates and invertebrates, suggesting that TADs associated with GRBs display unique genomic features. TADs which closely correspond with GRBs are termed ‘GRB–TADs’, and those that show no evidence of non-coding conservation ‘nonGRB–TADs’. Several features distinguish GRB–TADs from nonGRB–TADs; GRB–TADs are larger than nonGRB–TADs, gene-sparse and their target genes are expressed in a cell-type and tissue-specific manner. In contrast, nonGRB–TADs more often span regions of high gene density, and the strength of within-TAD interactions in them is consistently lower than in GRB–TADs (53). This may indicate a less defined or less consistent 3D organization across Hi-C experiments. Since strong and stable GRB–TADs are interspersed with less strongly interacting nonGRB–TADs there is an open possibility that the weaker TADs are simply regions between stronger TADs, which would mean that a stable 3D arrangement is not required in the absence of long-range regulation. At present, this is still a hypothesis (53), whose testing will require more high-resolution Hi-C data across different cell types and different species.

The observation that GRBs coincide with TADs around developmental genes puts an interesting twist on the question of co-regulation of genes within TADs. Since the GRB model predicts different expression profiles of target and bystander genes, with bystanders typically broadly expressed, the co-regulation of target and bystander genes is not expected. Harmston *et al.* examined several GRB loci for co-regulation and showed that the dynamic range of target gene expression is much wider than that of bystander genes (53). Moreover, on the limited number of loci, they show that GRB–TADs in different cell types switch between the two compartments identified by Hi-C: the A compartment (reported to be dominated by actively transcribing chromatin) and the B compartment (enriched for heterochromatin and other transcriptionally inactive regions). Remarkably, the activity state of the GRB target gene is the only one that predicts whether the GRB will be in the A or B compartment: the expression of bystander genes appears to change little between the two. While still a preliminary observation, this pattern is consistent with the GRB model of long-range regulation.

#### CNEs are an ancient feature of metazoan genomes

While the bulk of CNE research has focused on the identification and functional characterization of CNEs in human and other vertebrate genomes, it is clear that CNEs are not a vertebrate innovation as equivalent elements are detected in multiple metazoan lineages (32,54–58). Despite a lack of CNEs identifiable between each clade, some general features of CNEs are recapitulated in each case:

- CNEs occur in clusters around crucial regulators of early development such as *MEIS1* (59), *SHH* (60), *IRX3* (27) and around the orthologs of these genes in other lineages.
- CNEs have constrained genome organization and thus occur in GRBs in other metazoa (30,45).
- Finally, genes proposed as targets of CNE regulation are marked by broad polycomb repression in an inactive state (45).

The observation that CNEs are so prevalent and functionally similar within metazoan genomes indicates that they are an ancient and crucial feature of metazoan gene regulation.

#### Plant CNEs

Clusters of non-coding conservation also occur in genomes of higher plants (61–64), although their equivalence to metazoan CNEs is unclear. In plants, CNEs were found to cluster around genes involved in responses to hormonal stimuli, regulation of organ development (64–67) and flowering-time control (68). However, plant CNEs have so far not been shown to form GRB-like clusters, and much more work is needed to understand their distribution and roles.

### Sequence features of CNEs

**Figure 3.**
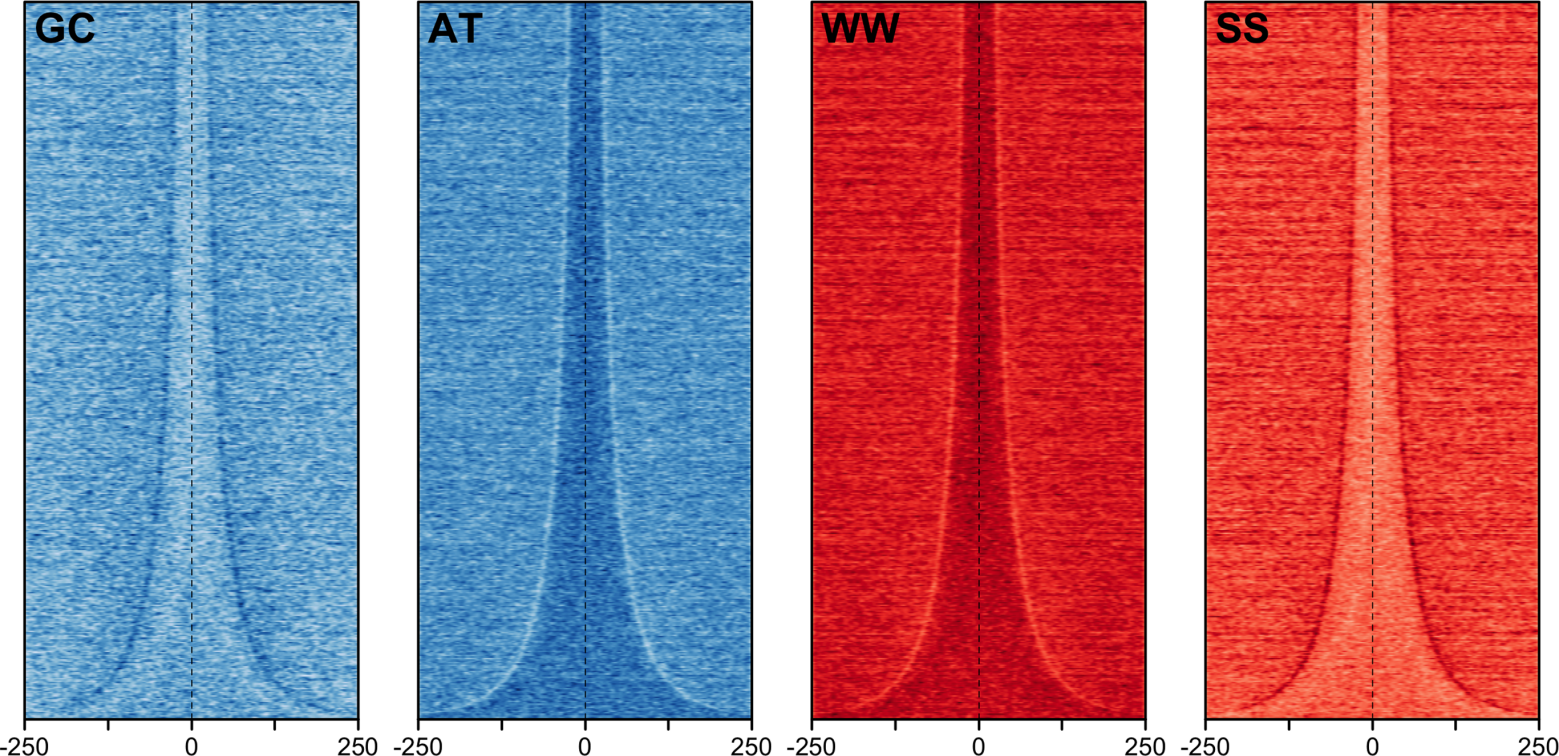
Sequence heatmaps showing dinucleotide content within and outside vertebrate CNEs. Plots are generated using *heatmaps* package (https://bioconductor.org/packages/release/bioc/html/heatmaps.html). CNEs which show sequence identity >98% for >50 nt between human and chicken are identified using *CNEr* (https://bioconductor.org/packages/release/bioc/html/CNEr.html). Sequences are ordered from shortest to longest on the *Y*-axis (aligned on the center) and *X*-axis shows distance in nucleotides from the center of each CNE.

Walter *et al.* analyzed the nucleotide composition of human and fugu CNEs, showing that they are AT-rich and often contain runs of identical nucleotides (69). This is in contrast to the flanking regions just outside their boundaries which exhibit a marked drop in AT content, forming a distinct composition pattern. In line with this, Chiang *et al.* have shown that vertebrate CNEs are enriched in TAATTA, which contains the core recognition motif (TAAT) for homeodomain DNA-binding factors (70). In Figure 3, we order vertebrate CNEs (identified using human/chicken whole-genome alignments) by width, and plot the enrichment of AT, GC, WW and SS dinucleotides. This clearly illustrates this boundary effect. As regions outside boundaries of CNEs are by definition mismatched, i.e. mutated sites in the alignment, the WW depletion at the boundaries of CNEs might be due to the higher mutability of CpG nucleotides. However, the latter does not explain why we find the same pattern in *Drosophila* where CpG methylation is absent. Importantly also, such GC-content transitions are known to occur at transcription boundaries and serve as genomic punctuation marks (71). In summary, it is still unclear why we find this pattern in CNEs.

#### CNEs overlap transcription factor binding sites (TFBS)

One of the suggested explanations for extreme non-coding conservation would be that CNEs constitute an ordered combination of overlapping TFBS (72,73). While there is clear evidence that CNEs are strongly enriched for overlapping TFBS (74,75), there is no evidence to suggest that this enrichment is higher for CNEs than for enhancers in general. Furthermore, it is unclear whether overlapping binding sites would be sufficient to explain extreme non-coding conservation, given promiscuity of binding sites and binding site divergence between species (76). A recent paper also proposed that CNEs are not under selective pressure as a whole DNA segment but are under various evolutionary constraints on the single nucleotide level (77), suggesting that overlapping TFBS likely do not account for the degree of conservation characteristic of CNEs.

#### Mining the distinguishing sequence features of CNEs

Two approaches have been presented to classify CNEs and distinguish them from other constrained elements within and between genomes: (i) N-gram graphs which combine neighborhood information (co-occurrence of substrings) with sequence compositional motifs (78) and (ii) logic alignment free which attempts to infer logic rules based on the underlying lexicon of sequences (79). Both approaches concluded that the most extremely conserved CNEs form a unique category bearing sequence features distinct from protein-coding exons. More sophisticated methods could be applied to CNE classification and deep learning, a variation of multi-layered artificial neural networks, is a promising candidate to elucidate potentially complex patterns within CNEs (80–82).

## CNEs AS PHYLOGENETIC MARKERS

**Figure 4.**
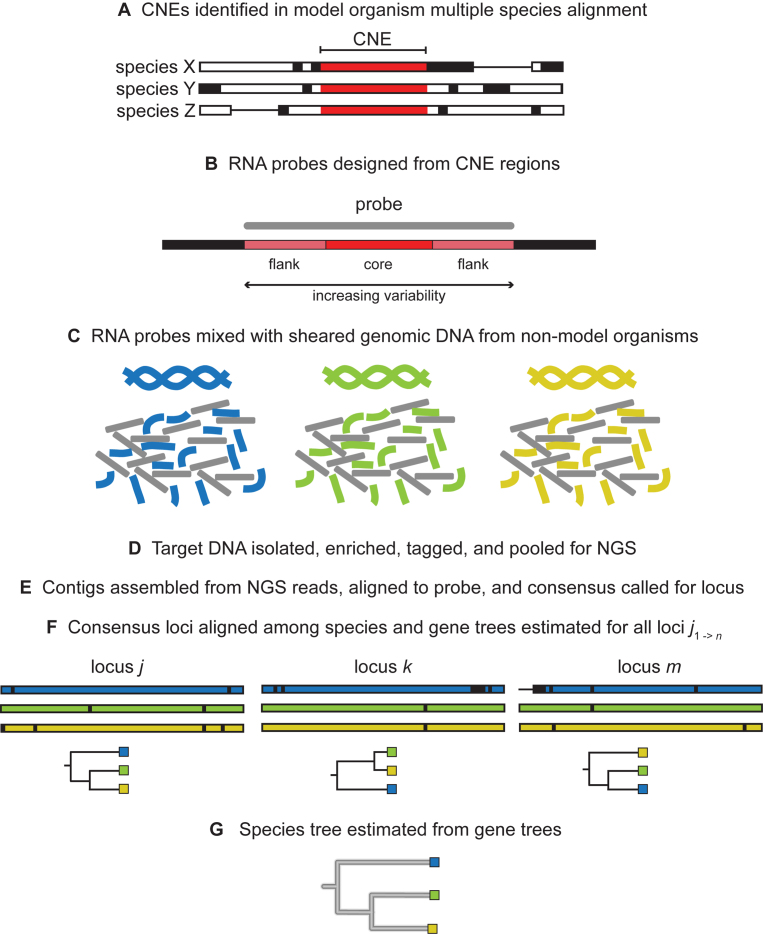
Methodology describing how CNEs are utilized as markers for constructing phylogenies (adopted and modified with permission from Brant Faircloth and John McCormack). Panels (**A**-**G**) describe the steps for constructing phylogenies starting from CNE sequences to generating species trees.

The deep sequence conservation of CNEs across phylogenies makes them particularly useful for the elucidation of evolutionary relationships. Using CNEs as the anchor points for targeted DNA enrichment and sequencing, Faircloth *et al.* recovered the established primate phylogeny (83) and McCormack *et al.* resolved the placental mammal phylogeny (84). As a further proof of concept, this approach was applied to 32 arachnids, again producing a highly resolved arachnid phylogeny consistent with transcriptome-based phylogenetic analyses. Due to the increasing variability of the sequence flanking the ‘core’ CNE region, the authors were also able to generate accurate phylogenies of the spider, scorpion and harvestman orders, demonstrating the utility of this method for shallower taxonomic scales (85,86). CNEs as probes have also proven useful in the case of ancient and degraded DNA (87). An overview of the workflow for using CNEs as phylogenomics markers is provided in Figure 4.

This method is implemented in the software package PHYLUCE (88). It is particularly useful for phylogenomic analysis of non-model organisms, as the extreme conservation of CNEs allows for targeted sequencing of informative loci without a complete reference genome. The ‘core’ regions of CNEs alone are sufficient to recapitulate gene trees, as demonstrated by Davies *et al.* (89), and have recently been considered for resolving nodes that are difficult to place in the eutherian tree (90). For a comprehensive review on CNEs as a tool in phylogenomics, see Edwards *et al.* (91).

## DISEASES ASSOCIATED WITH NON-CODING CONSERVATION

Mutations in CNEs have been established as causal for a number of diseases. Single point mutations are associated with malformations, including *Pierre Robin syndrome* (92), *cleft lip* (93), *holoprosencephaly* (36,94), *preaxial polydactyly* (35,37,95) and *Hirschsprung* disease (96). A single nucleotide variant associated with *IRX1, IRX2* and *IRX4*, located within a CNE, was also recently found to be involved in the pathogenesis of *kyphoscoliosis* (97). Beyond malformations, variations within CNEs can even be linked to complex behavioral or neurological disorders, evident from research linking cases of attention-deficit/hyperactivity disorder (98), autism (99) and restless leg syndrome (100) to single point mutations within CNEs.

Several diseases have been linked with duplications of CNEs, e.g. *brachydactyly A2* (101) and *brachydactyly-anonychia* (102). Copy-number variations of the Indian Hedgehog region involving CNEs are related to *syndactyly* and *craniosynostosis* (103). Translocation of a CNE has been implicated in the aetiology of aniridia (104). Deafness (105), Leri-Weill dyschondrosteosis (25), blepharophimosis syndrome (106,107) and Waardenburg syndrome type 4 (108) are well-known cases of pathologies that are associated with deletions of CNEs. These findings further highlight and establish the role of CNEs in neurodevelopmental diseases. For a comprehensive review on this subject, see Amiel *et al.* (109).

### Large-scale CNE deletions without visible phenotype

Despite strong evidence that the majority of CNEs play crucial roles in development, two early studies found that deletions of entire CNE-rich loci produced no detectable phenotypic changes in mouse models (110,111). However, it may be the case that either the phenotypes were difficult to detect in the tested experimental context, or that the tested elements were not deeply conserved. More elaborate methodologies are required to study CNE loss in greater detail. Towards this direction, Dickel and colleagues recently used CRISPR to knockout four CNEs near *ARX*, a gene with roles in sexual and brain development (112). At first, removing these elements individually or in pairs resulted in seemingly unaffected mice. A closer inspection of the brains of the knockout mice, however, revealed an atypical number of neurons or a diminished hippocampus, both of which were more pronounced in the double knockout mice. This supports the idea that a CNE phenotype might be context-specific, having little effect on mice in laboratory conditions but potentially detrimental in the wild.

### Transcribed ultraconserved regions (T-UCRs)

Other than congenital abnormalities associated mainly with development, there are confirmed roles for CNEs in cancer. Calin *et al.* compared the transcription levels of the 481 ultraconserved regions from Bejerano *et al.* (10), and found that 93% of of those regions were transcribed over background in at least one of the tested normal human tissues. They named those elements transcribed ultraconserved regions (T-UCRs), and demonstrated that CNE transcriptional profiles could be utilized in order to differentiate carcinomas from leukemias (113). Since then, roles of T-UCRs have been investigated in hepatocellular carcinoma (114), prostate cancer (115,116), colorectal carcinoma (113,117), neuroblastoma (118,119), Barrett’s esophagus (120) and bladder cancer (121). In addition, reducing the overexpression of a T-UCR (uc.261) has been suggested as a therapeutic intervention for patients with Crohn’s disease (122).

At the level of GRBs, highly conserved elements that serve as long-range regulatory input for the TF genes *HHEX, SOX4* and *IRX3* were found to be associated with type 2 diabetes and obesity (123–125). Additional cases where GRBs are implicated in human diseases may be found in a review by Navratilova and Becker (126).

Summarizing this section, disruption of CNEs can contribute to the onset of severe diseases mainly associated with development and cancer. We anticipate more examples will be discovered in the future, especially now that it has been established that the majority of GWAS SNPs lie in the non-coding part of our genomes (127,128). It is possible that loss of even the most conserved CNEs is not guaranteed to result in visible phenotypes, emphasizing how much we still have to learn about extreme non-coding conservation.

## EMERGENCE AND EVOLUTIONARY DYNAMICS OF CONSERVED NON-CODING ELEMENTS

The structural and implied functional equivalence of CNEs across vastly different realms of life, along with their extreme levels of sequence conservation, suggest that CNEs perform an ancient and irreplaceable function in genomes of multicellular eukaryotes. The emergence and maintenance of CNEs however remain poorly understood and no current theories can satisfactorily explain the source of selective pressure capable of maintaining such extreme levels of conservation (129).

Initial speculations that CNEs were not a product of negative selection, but simply genomic loci with a low local mutation rate, were dispelled by evidence that CNEs exhibit traits associated with sequences under purifying selection–namely very low derived allele frequencies, indicating evolutionary suppression of variation within these elements (130–132). This finding was corroborated in 2014 using 1000 Genomes human variation data (133).

### Emergence

CNEs are readily recruited *de novo* from a diverse range of genomic sequences, an observation which is reflected by the general lack of similarity between CNEs on a sequence level. There is evidence for CNE recruitment from introns (10,54,56), transposable elements (TE) (134,135) and ancient repeats (136). There also exist several examples of CNEs which have been recruited from parts of exons (137,138). While GRBs are depleted of TEs, it seems that TEs that have been retained have significantly contributed to lineage-specific CNE evolution (139). Of the CNEs arising since split of eutheria and marsupials, 16% contain recognizable TEs. This is in contrast to 0.7% of the CNEs which had an orthologous marsupial CNE. These TE-derived, lineage-specific CNEs may underlie some of the innovative features responsible for eutherian-specific morphology and neural development.

CNE recruitment varies across lineages, with primates appearing to gain CNEs at particularly high rates (140). This recruitment tends to be enriched around different genes in different lineages, although CNEs are also gained around a conserved core of developmental genes (141). Furthermore, Lowe *et al.* (142) have proposed that there have been three distinct periods of regulatory innovation during vertebrate evolution: CNEs appear to have been preferentially recruited around genes that encode for TFs and key developmental regulators during early vertebrate evolution, then to cell signaling genes, and then to genes involved in post-translational modifications during placental mammal evolution. These patterns underline the importance of CNE recruitment in shaping vertebrate evolution. Further, it has been suggested that following the initial recruitment of a CNE, it’s flanking sequences could be co-opted to regulatory function over time in a lineage-specific manner (143). Under this model, the recruited flanking sequence would increase the modularity and complexity of the overall regulation by the element. This could explain that the core conserved regions of vertebrate enhancers are often sufficient to drive gene expression in reporter assays (144). However, these observations could also be explained by flanking sequences being under a relatively lower selection pressure than the core CNE region.

Taken together, the literature suggests that CNEs are recruited from any existing genomic sequence within the reach of the target gene if it contributes an advantageous alteration in gene expression. Furthermore, the alteration of key developmental genes’ expression can and does underlie the basis of many lineage-specific traits. Still, however, the mechanism by which CNE recruitment occurs remains unknown.

### CNE modifications and losses

Despite the high degree of sequence and functional maintenance typical of CNEs, substitutions and deletions still occur, albeit at a much reduced rate. Such changes can be without apparent phenotype, but equally can be disruptive, and likely underlie many lineage-specific traits.

Losses of individual CNEs can be sufficient for alterations to anatomical structures. Deletions are thought to underlie penile spine loss (145) and foot digit shortening in humans (146). More extensive changes have been observed in other animals: snake limblessness, for example, is associated with partial and complete deletions of CNEs that regulate limb development genes (147–150). For example, a vertebrate conserved *SHH* enhancer has a few substitutions in snakes with vestigial hindlimbs, but a short deletion and multiple changes in snakes that have undergone complete limb loss (148). Interestingly, this ZRS enhancer is the same element implicated in human polydactyly. In stickleback, the deletion of a CNE, regulating the developmental gene *PITX1*, leads to pelvic reduction. The loss of this element, which may be beneficial due to reduced predation and calcium availability in freshwater environments, has occurred multiple times in independent freshwater populations, with strong evidence that *PITX1* regulatory mutations are under positive selection in these populations (151). CNEs are even recurrently lost across species, with Hiller *et al.* discovering hundreds of CNEs independently lost in more than one mammal (152). A recent study demonstrated that many such losses could be linked to common anatomical changes. Those included an independent loss of an element proximal to *EGR2*, a TF linked to forelimb morphology, in manatees and dolphins. The deletions are postulated to play a role in elbow structure modifications common to both species (153). Additionally, the vast phenotypic diversity of teleost fish has been hypothesized to be related to large-scale loss of and increased substitution rates in ancestral vertebrate CNEs (154). The recent whole genome sequencing of one of the more morphologically distinct teleosts, the seahorse (*Hippocampus comes*), revealed a high degree of CNE loss compared to other teleost fish. Many of these CNEs cluster around key retained developmental genes, and may have contributed to the extensive morphological changes in seahorses (155).

As new elements are regularly recruited, a subset will have near-equivalent functions to extant CNEs, and thus could potentially replace them. This appears to be the case for nPE1 and nPE2, mammalian enhancers of *POMC* derived from TE (156). These elements have likely replaced ancestral enhancers, which are lost in mammals but still maintained in other vertebrates (157). The CNE turnover model posits that in the long term no sequences are indispensable, and that such *turnover* of elements may be quite common (129). This would explain why expression patterns are often preserved across species despite large changes in *cis*-regulation (158).

Several thousand CNEs have undergone bursts of lineage-specific positive selection in humans (159–164). These elements, referred to as human accelerated regions, are highly conserved in most mammals and often in other vertebrates, but have rapidly accumulated substitutions in humans. A number of these regions have been tested using transgenic mouse models, comparing how the human sequence drives gene expression compared to the equivalent in chimpanzee. This has demonstrated that some, often very highly conserved, CNEs have divergent function in humans, seemingly contributing to several human-specific traits, including bipedalism and increased brain size (165,166).

## PERSPECTIVES

Most CNEs act as developmental enhancers, however, this does not explain the extreme levels of their conservation. Methodological advances for studying those elements *in vivo* are necessary: recently proposed genome-wide editing techniques for large-scale interrogation of regulatory elements (167,168) could prove promising towards addressing the role of CNEs on an individual or on a per-GRB basis.

We conclude by outlining questions, the answers to which may further our understanding of CNEs and their function:

- *What is the origin of selection pressure on CNEs?* CNEs that form clusters around developmental genes share the unknown source of purifying selection. According to this criterion, they are not a set of elements with different function. However, we still lack understanding on what is being selected for in CNEs and how.
- *How ancient is the mode of regulation that includes CNEs?* There are CNE-like elements in plants and some fungi, which cluster around genes involved in morphogenesis. We still do not know where in evolution the phenomenon of extreme non-coding conservation emerged, but the available evidence points to early emergence in multicellular eukaryotes.
- *Are CNEs that belong to the same GRB more similar to each other compared to CNEs found in other GRBs?* Most CNEs are single copy elements (10) although there is a subset of non-unique CNEs in the human genome that appear to be the result of whole genome duplication events (169). We currently do not know what are the sequence properties of CNEs within different GRBs and whether different sequence features within CNEs play fundamental roles in the regulation of corresponding target gene(s).
- *What is the sequence grammar of extreme non-coding conservation?* Our grasp of the importance of spacing, orientation and copy number of TFBS for CNEs function remains rudimentary. It is currently unknown whether the underlying organization of consensus motifs within CNEs could have potential impact on their functionalities.
- *What is the role (if any) for CNEs in the folding, establishment or maintenance of TADs?* TAD structures are lost during mitosis, indicating that TADs must consistently refold after each cell division. The principles that guide TAD folding and maintenance are still largely unknown. Given the correspondence between GRBs and TADs, it would be tempting to investigate the possible role of CNEs in TAD formation.
- *What is the role of CNEs in disease pathogenesis?* Recent studies have demonstrated that when elements that define TAD boundaries are disrupted, distinct developmental disorders and cancer arise (170–173). Given the role of CNEs in diseases and the correspondence between GRBs and TADs, it will be interesting to explore how enhancer–promoter interactions are perturbed in various diseases, in the context of GRBs.

## Supplementary Material

Supplementary Data

## References

[1] Yaffe D., Nudel U., Mayer Y., Neuman S. Highly conserved sequences in the 3’ untranslated region of mRNAs coding for homologous proteins in distantly related species. Nucleic Acids Res. 1985; 13:3723–3737. 4011440 10.1093/nar/13.10.3723 PMC341269

[2] Lemaire C., Heilig R., Mandel J.L. The chicken dystrophin cDNA: striking conservation of the C-terminal coding and 3′ untranslated regions between man and chicken. EMBO J. 1988; 7:4157–4162. 3072195 10.1002/j.1460-2075.1988.tb03311.x PMC455126

[3] Hraba-Renevey S., Kress M. Expression of a mouse replacement histone H3.3 gene with a highly conserved 3′ noncoding region during SV40- and polyoma-induced Go to S-phase transition. Nucleic Acids Res. 1989; 17:2449–2461. 2470025 10.1093/nar/17.7.2449 PMC317635

[4] Kajimoto Y., Rotwein P. Structure of the chicken insulin-like growth factor I gene reveals conserved promoter elements. J. Biol. Chem. 1991; 266:9724–9731. 2033062

[5] Rouault J.P., Samarut C., Duret L., Tessa C., Samarut J., Magaud J.P. Sequence analysis reveals that the BTG1 anti-proliferative gene is conserved throughout evolution in its coding and 3′ non-coding regions. Gene. 1993; 129:303–306. 8325512 10.1016/0378-1119(93)90284-a

[6] Duret L., Dorkeld F., Gautier C. Strong conservation of non-coding sequences during vertebrates evolution: potential involvement in post-transcriptional regulation of gene expression. Nucleic Acids Res. 1993; 21:2315–2322. 8506129 10.1093/nar/21.10.2315 PMC309526

[7] Hardison R.C., Oeltjen J., Miller W. Long human-mouse sequence alignments reveal novel regulatory elements: a reason to sequence the mouse genome. Genome Res. 1997; 7:959–966. 9331366 10.1101/gr.7.10.959

[8] Hardison R.C. Conserved noncoding sequences are reliable guides to regulatory elements. Trends Genet. 2000; 16:369–372. 10973062 10.1016/s0168-9525(00)02081-3

[9] Loots G.G., Locksley R.M., Blankespoor C.M., Wang Z.E., Miller W., Rubin E.M., Frazer K.A. Identification of a coordinate regulator of interleukins 4, 13, and 5 by cross-species sequence comparisons. Science. 2000; 288:136–140. 10753117 10.1126/science.288.5463.136

[10] Bejerano G., Pheasant M., Makunin I., Stephen S., Kent W.J., Mattick J.S., Haussler D. Ultraconserved elements in the human genome. Science. 2004; 304:1321–1325. 15131266 10.1126/science.1098119

[11] Sandelin A., Bailey P., Bruce S., Engström P.G., Klos J.M., Wasserman W.W., Ericson J., Lenhard B. Arrays of ultraconserved non-coding regions span the loci of key developmental genes in vertebrate genomes. BMC Genomics. 2004; 5:99. 15613238 10.1186/1471-2164-5-99 PMC544600

[12] Woolfe A., Goodson M., Goode D.K., Snell P., McEwen G.K., Vavouri T., Smith S.F., North P., Callaway H., Kelly K. Highly conserved non-coding sequences are associated with vertebrate development. PLoS Biol. 2005; 3:e7. 15630479 10.1371/journal.pbio.0030007 PMC526512

[13] Engström P.G., Fredman D., Lenhard B. Ancora: a web resource for exploring highly conserved noncoding elements and their association with developmental regulatory genes. Genome Biol. 2008; 9:R34. 18279518 10.1186/gb-2008-9-2-r34 PMC2374709

[14] Dousse A., Junier T., Zdobnov E.M. CEGA–a catalog of conserved elements from genomic alignments. Nucleic Acids Res. 2016; 44:D96–D100. 26527719 10.1093/nar/gkv1163 PMC4702837

[15] Persampieri J., Ritter D.I., Lees D., Lehoczky J., Li Q., Guo S., Chuang J.H. cneViewer: a database of conserved non-coding elements for studies of tissue-specific gene regulation. Bioinformatics. 2008; 24:2418–2419. 18718943 10.1093/bioinformatics/btn443 PMC2562007

[16] Woolfe A., Goode D.K., Cooke J., Callaway H., Smith S., Snell P., McEwen G.K., Elgar G. CONDOR: a database resource of developmentally associated conserved non-coding elements. BMC Dev. Biol. 2007; 7:100. 17760977 10.1186/1471-213X-7-100 PMC2020477

[17] Lomonaco V., Martoglia R., Mandreoli F., Anderlucci L., Emmett W., Bicciato S., Taccioli C. UCbase 2.0: ultraconserved sequences database (2014 update). Database (Oxford). 2014; 2014:bau062. 24951797 10.1093/database/bau062 PMC4064129

[18] Dimitrieva S., Bucher P. UCNEbase–a database of ultraconserved non-coding elements and genomic regulatory blocks. Nucleic Acids Res. 2013; 41:D101–D109. 23193254 10.1093/nar/gks1092 PMC3531063

[19] Visel A., Minovitsky S., Dubchak I., Pennacchio L.A. VISTA Enhancer Browser–a database of tissue-specific human enhancers. Nucleic Acids Res. 2007; 35:D88–D892. 17130149 10.1093/nar/gkl822 PMC1716724

[20] Aparicio S., Morrison A., Gould A., Gilthorpe J., Chaudhuri C., Rigby P., Krumlauf R., Brenner S. Detecting conserved regulatory elements with the model genome of the Japanese puffer fish, Fugu rubripes. Proc. Natl. Acad. Sci. U.S.A. 1995; 92:1684–1688. 7878040 10.1073/pnas.92.5.1684 PMC42584

[21] Visel A., Prabhakar S., Akiyama J.A., Shoukry M., Lewis K.D., Holt A., Plajzer-Frick I., Afzal V., Rubin E.M., Pennacchio L.A. Ultraconservation identifies a small subset of extremely constrained developmental enhancers. Nat. Genet. 2008; 40:158–160. 18176564 10.1038/ng.2007.55 PMC2647775

[22] Pennacchio L.A., Ahituv N., Moses A.M., Prabhakar S., Nobrega M.A., Shoukry M., Minovitsky S., Dubchak I., Holt A., Lewis K.D. In vivo enhancer analysis of human conserved non-coding sequences. Nature. 2006; 444:499–502. 17086198 10.1038/nature05295

[23] Shen Y., Yue F., McCleary D.F., Ye Z., Edsall L., Kuan S., Wagner U., Dixon J., Lee L., Lobanenkov V.V. A map of the cis-regulatory sequences in the mouse genome. Nature. 2012; 488:116–120. 22763441 10.1038/nature11243 PMC4041622

[24] Nobrega M.A., Ovcharenko I., Afzal V., Rubin E.M. Scanning human gene deserts for long-range enhancers. Science. 2003; 302:413. 14563999 10.1126/science.1088328

[25] Sabherwal N., Bangs F., Röth R., Weiss B., Jantz K., Tiecke E., Hinkel G.K., Spaich C., Hauffa B.P., van der Kamp H. Long-range conserved non-coding SHOX sequences regulate expression in developing chicken limb and are associated with short stature phenotypes in human patients. Hum. Mol. Genet. 2007; 16:210–222. 17200153 10.1093/hmg/ddl470

[26] Maas S.A., Suzuki T., Fallon J.F. Identification of spontaneous mutations within the long-range limb-specific Sonic hedgehog enhancer (ZRS) that alter Sonic hedgehog expression in the chicken limb mutants oligozeugodactyly and silkie breed. Dev. Dyn. 2011; 240:1212–1222. 21509895 10.1002/dvdy.22634 PMC3464974

[27] de la Calle-Mustienes E., Feijóo C.G., Manzanares M., Tena J.J., Rodríguez-Seguel E., Letizia A., Allende M.L., Gómez-Skarmeta J.L. A functional survey of the enhancer activity of conserved non-coding sequences from vertebrate Iroquois cluster gene deserts. Genome Res. 2005; 15:1061–1072. 16024824 10.1101/gr.4004805 PMC1182218

[28] Kikuta H., Fredman D., Rinkwitz S., Lenhard B., Becker T.S. Retroviral enhancer detection insertions in zebrafish combined with comparative genomics reveal genomic regulatory blocks - a fundamental feature of vertebrate genomes. Genome Biol. 2007; 8(Suppl. 1):S4. 18047696 10.1186/gb-2007-8-s1-s4 PMC2106839

[29] Shin J.T., Priest J.R., Ovcharenko I., Ronco A., Moore R.K., Burns C.G., MacRae C.A. Human-zebrafish non-coding conserved elements act in vivo to regulate transcription. Nucleic Acids Res. 2005; 33:5437–5445. 16179648 10.1093/nar/gki853 PMC1236720

[30] Kikuta H., Laplante M., Navratilova P., Komisarczuk A.Z., Engström P.G., Fredman D., Akalin A., Caccamo M., Sealy I., Howe K. Genomic regulatory blocks encompass multiple neighboring genes and maintain conserved synteny in vertebrates. Genome Res. 2007; 17:545–555. 17387144 10.1101/gr.6086307 PMC1855176

[31] Sanges R., Kalmar E., Claudiani P., D’Amato M., Muller F., Stupka E. Shuffling of cis-regulatory elements is a pervasive feature of the vertebrate lineage. Genome Biol. 2006; 7:R56. 16859531 10.1186/gb-2006-7-7-r56 PMC1779573

[32] Sanges R., Hadzhiev Y., Gueroult-Bellone M., Roure A., Ferg M., Meola N., Amore G., Basu S., Brown E.R., De Simone M. Highly conserved elements discovered in vertebrates are present in non-syntenic loci of tunicates, act as enhancers and can be transcribed during development. Nucleic Acids Res. 2013; 41:3600–3618. 23393190 10.1093/nar/gkt030 PMC3616699

[33] Blow M.J., McCulley D.J., Li Z., Zhang T., Akiyama J.A., Holt A., Plajzer-Frick I., Shoukry M., Wright C., Chen F. ChIP-Seq identification of weakly conserved heart enhancers. Nat. Genet. 2010; 42:806–810. 20729851 10.1038/ng.650 PMC3138496

[34] May D., Blow M.J., Kaplan T., McCulley D.J., Jensen B.C., Akiyama J.A., Holt A., Plajzer-Frick I., Shoukry M., Wright C. Large-scale discovery of enhancers from human heart tissue. Nat. Genet. 2011; 44:89–93. 22138689 10.1038/ng.1006 PMC3246570

[35] Lettice L.A., Heaney S.J.H., Purdie L.A., Li L., de Beer P., Oostra B.A., Goode D., Elgar G., Hill R.E., de Graaff E. A long-range Shh enhancer regulates expression in the developing limb and fin and is associated with preaxial polydactyly. Hum. Mol. Genet. 2003; 12:1725–1735. 12837695 10.1093/hmg/ddg180

[36] Jeong Y., Leskow F.C., El-Jaick K., Roessler E., Muenke M., Yocum A., Dubourg C., Li X., Geng X., Oliver G. Regulation of a remote Shh forebrain enhancer by the Six3 homeoprotein. Nat. Genet. 2008; 40:1348–1353. 18836447 10.1038/ng.230 PMC2648611

[37] Lettice L.A., Hill R.E. Preaxial polydactyly: a model for defective long-range regulation in congenital abnormalities. Curr. Opin. Genet. Dev. 2005; 15:294–300. 15917205 10.1016/j.gde.2005.04.002

[38] Sharpe J., Lettice L., Hecksher-Sorensen J., Fox M., Hill R., Krumlauf R. Identification of sonic hedgehog as a candidate gene responsible for the polydactylous mouse mutant Sasquatch. Curr. Biol. 1999; 9:97–100. 10021368 10.1016/s0960-9822(99)80022-0

[39] Turner E.E., Cox T.C. Genetic evidence for conserved non-coding element function across species-the ears have it. Front. Physiol. 2014; 5:7. 24478720 10.3389/fphys.2014.00007 PMC3896894

[40] Nolte M.J., Wang Y., Deng J.M., Swinton P.G., Wei C., Guindani M., Schwartz R.J., Behringer R.R. Functional analysis of limb transcriptional enhancers in the mouse. Evol. Dev. 2014; 16:207–223. 24920384 10.1111/ede.12084 PMC4130292

[41] Polychronopoulos D., Sellis D., Almirantis Y. Conserved noncoding elements follow power-law-like distributions in several genomes as a result of genome dynamics. PLoS One. 2014; 9:e95437. 24787386 10.1371/journal.pone.0095437 PMC4008492

[42] Salerno W., Havlak P., Miller J. Scale-invariant structure of strongly conserved sequence in genomic intersections and alignments. Proc. Natl. Acad. Sci. U.S.A. 2006; 103:13121–13125. 16924100 10.1073/pnas.0605735103 PMC1559763

[43] Polychronopoulos D., Athanasopoulou L., Almirantis Y. Fractality and entropic scaling in the chromosomal distribution of conserved noncoding elements in the human genome. Gene. 2016; 584:148–160. 26899868 10.1016/j.gene.2016.02.022

[44] Kim S.Y., Pritchard J.K. Adaptive evolution of conserved noncoding elements in mammals. PLoS Genet. 2007; 3:1572–1586. 17845075 10.1371/journal.pgen.0030147 PMC1971121

[45] Engström P.G., Ho Sui S.J., Drivenes O., Becker T.S., Lenhard B. Genomic regulatory blocks underlie extensive microsynteny conservation in insects. Genome Res. 2007; 17:1898–1908. 17989259 10.1101/gr.6669607 PMC2099597

[46] Akalin A., Fredman D., Arner E., Dong X., Bryne J.C., Suzuki H., Daub C.O., Hayashizaki Y., Lenhard B. Transcriptional features of genomic regulatory blocks. Genome Biol. 2009; 10:R38. 19374772 10.1186/gb-2009-10-4-r38 PMC2688929

[47] Nora E.P., Lajoie B.R., Schulz E.G., Giorgetti L., Okamoto I., Servant N., Piolot T., van Berkum N.L., Meisig J., Sedat J. Spatial partitioning of the regulatory landscape of the X-inactivation centre. Nature. 2012; 485:381–385. 22495304 10.1038/nature11049 PMC3555144

[48] Dixon J.R., Selvaraj S., Yue F., Kim A., Li Y., Shen Y., Hu M., Liu J.S., Ren B. Topological domains in mammalian genomes identified by analysis of chromatin interactions. Nature. 2012; 485:376–380. 22495300 10.1038/nature11082 PMC3356448

[49] Acemel R.D., Maeso I., Gómez-Skarmeta J.L. Topologically associated domains: a successful scaffold for the evolution of gene regulation in animals. Wiley Interdiscip. Rev. Dev. Biol. 2017; 6:doi:10.1002/wdev.265. 10.1002/wdev.265 28251841

[50] Rao S.S.P., Huntley M.H., Durand N.C., Stamenova E.K., Bochkov I.D., Robinson J.T., Sanborn A.L., Machol I., Omer A.D., Lander E.S. A 3D map of the human genome at kilobase resolution reveals principles of chromatin looping. Cell. 2014; 159:1665–1680. 25497547 10.1016/j.cell.2014.11.021 PMC5635824

[51] Dixon J.R., Jung I., Selvaraj S., Shen Y., Antosiewicz-Bourget J.E., Lee A.Y., Ye Z., Kim A., Rajagopal N., Xie W. Chromatin architecture reorganization during stem cell differentiation. Nature. 2015; 518:331–336. 25693564 10.1038/nature14222 PMC4515363

[52] Vietri Rudan M., Barrington C., Henderson S., Ernst C., Odom D.T., Tanay A., Hadjur S. Comparative Hi-C reveals that CTCF underlies evolution of chromosomal domain architecture. Cell Rep. 2015; 10:1297–1309. 25732821 10.1016/j.celrep.2015.02.004 PMC4542312

[53] Harmston N., Ing-Simmons E., Tan G., Perry M., Merkenschlager M., Lenhard B. Topologically associating domains are ancient features that coincide with Metazoan clusters of extreme noncoding conservation. Nat. Commun. 2017; 8:441. 28874668 10.1038/s41467-017-00524-5 PMC5585340

[54] Glazov E.A., Pheasant M., McGraw E.A., Bejerano G., Mattick J.S. Ultraconserved elements in insect genomes: a highly conserved intronic sequence implicated in the control of homothorax mRNA splicing. Genome Res. 2005; 15:800–808. 15899965 10.1101/gr.3545105 PMC1142470

[55] Vavouri T., Walter K., Gilks W.R., Lehner B., Elgar G. Parallel evolution of conserved non-coding elements that target a common set of developmental regulatory genes from worms to humans. Genome Biol. 2007; 8:R15. 17274809 10.1186/gb-2007-8-2-r15 PMC1852409

[56] Siepel A., Bejerano G., Pedersen J.S., Hinrichs A.S., Hou M., Rosenbloom K., Clawson H., Spieth J., Hillier L.W., Richards S. Evolutionarily conserved elements in vertebrate, insect, worm, and yeast genomes. Genome Res. 2005; 15:1034–1050. 16024819 10.1101/gr.3715005 PMC1182216

[57] Irvine S.Q. Study of cis-regulatory elements in the Ascidian Ciona intestinalis. Curr. Genomics. 2013; 14:56–67. 23997651 10.2174/138920213804999192 PMC3580780

[58] Clarke S.L., VanderMeer J.E., Wenger A.M., Schaar B.T., Ahituv N., Bejerano G. Human developmental enhancers conserved between deuterostomes and protostomes. PLoS Genet. 2012; 8:e1002852. 22876195 10.1371/journal.pgen.1002852 PMC3410860

[59] Royo J.L., Bessa J., Hidalgo C., Fernández-Miñán A., Tena J.J., Roncero Y., Gómez-Skarmeta J.L., Casares F. Identification and analysis of conserved cis-regulatory regions of the MEIS1 gene. PLoS One. 2012; 7:e33617. 22448256 10.1371/journal.pone.0033617 PMC3308983

[60] Anderson E., Devenney P.S., Hill R.E., Lettice L.A. Mapping the Shh long-range regulatory domain. Development. 2014; 141:3934–3943. 25252942 10.1242/dev.108480 PMC4197689

[61] Kritsas K., Wuest S.E., Hupalo D., Kern A.D., Wicker T., Grossniklaus U. Computational analysis and characterization of UCE-like elements (ULEs) in plant genomes. Genome Res. 2012; 22:2455–2466. 22987666 10.1101/gr.129346.111 PMC3514675

[62] Guo H., Moose S.P. Conserved noncoding sequences among cultivated cereal genomes identify candidate regulatory sequence elements and patterns of promoter evolution. Plant Cell. 2003; 15:1143–1158. 12724540 10.1105/tpc.010181 PMC153722

[63] Lockton S., Gaut B.S. Plant conserved non-coding sequences and paralogue evolution. Trends Genet. 2005; 21:60–65. 15680516 10.1016/j.tig.2004.11.013

[64] Inada D.C., Bashir A., Lee C., Thomas B.C., Ko C., Goff S.A., Freeling M. Conserved noncoding sequences in the grasses. Genome Res. 2003; 13:2030–2041. 12952874 10.1101/gr.1280703 PMC403677

[65] Hettiarachchi N., Kryukov K., Sumiyama K., Saitou N. Lineage-specific conserved noncoding sequences of plant genomes: their possible role in nucleosome positioning. Genome Biol. Evol. 2014; 6:2527–2542. 25364802 10.1093/gbe/evu188 PMC4202324

[66] Burgess D., Freeling M. The most deeply conserved noncoding sequences in plants serve similar functions to those in vertebrates despite large differences in evolutionary rates. Plant Cell. 2014; 26:946–961. 24681619 10.1105/tpc.113.121905 PMC4001403

[67] Haudry A., Platts A.E., Vello E., Hoen D.R., Leclercq M., Williamson R.J., Forczek E., Joly-Lopez Z., Steffen J.G., Hazzouri K.M. An atlas of over 90,000 conserved noncoding sequences provides insight into crucifer regulatory regions. Nat. Genet. 2013; 45:891–898. 23817568 10.1038/ng.2684

[68] Salvi S., Sponza G., Morgante M., Tomes D., Niu X., Fengler K.A., Meeley R., Ananiev E.V., Svitashev S., Bruggemann E. Conserved noncoding genomic sequences associated with a flowering-time quantitative trait locus in maize. Proc. Natl. Acad. Sci. U.S.A. 2007; 104:11376–11381. 17595297 10.1073/pnas.0704145104 PMC2040906

[69] Walter K., Abnizova I., Elgar G., Gilks W.R. Striking nucleotide frequency pattern at the borders of highly conserved vertebrate non-coding sequences. Trends Genet. 2005; 21:436–440. 15979195 10.1016/j.tig.2005.06.003

[70] Chiang C.W.K., Derti A., Schwartz D., Chou M.F., Hirschhorn J.N., Wu C.-T. Ultraconserved elements: analyses of dosage sensitivity, motifs and boundaries. Genetics. 2008; 180:2277–2293. 18957701 10.1534/genetics.108.096537 PMC2600958

[71] Zhang L., Kasif S., Cantor C.R., Broude N.E. GC/AT-content spikes as genomic punctuation marks. Proc. Natl. Acad. Sci. U.S.A. 2004; 101:16855–16860. 15548610 10.1073/pnas.0407821101 PMC534751

[72] Levy S., Hannenhalli S., Workman C. Enrichment of regulatory signals in conserved non-coding genomic sequence. Bioinformatics. 2001; 17:871–877. 11673231 10.1093/bioinformatics/17.10.871

[73] Loots G.G., Ovcharenko I., Pachter L., Dubchak I., Rubin E.M. rVista for comparative sequence-based discovery of functional transcription factor binding sites. Genome Res. 2002; 12:832–839. 11997350 10.1101/gr.225502 PMC186580

[74] Viturawong T., Meissner F., Butter F., Mann M. A DNA-centric protein interaction map of ultraconserved elements reveals contribution of transcription factor binding hubs to conservation. Cell Rep. 2013; 5:531–545. 24139795 10.1016/j.celrep.2013.09.022

[75] Warnefors M., Hartmann B., Thomsen S., Alonso C.R. Combinatorial gene regulatory functions underlie ultraconserved elements in drosophila. Mol. Biol. Evol. 2016; 33:2294–2306. 27247329 10.1093/molbev/msw101 PMC4989106

[76] Schmidt D., Wilson M.D., Ballester B., Schwalie P.C., Brown G.D., Marshall A., Kutter C., Watt S., Martinez-Jimenez C.P., Mackay S. Five-vertebrate ChIP-seq reveals the evolutionary dynamics of transcription factor binding. Science. 2010; 328:1036–1040. 20378774 10.1126/science.1186176 PMC3008766

[77] Silla T., Kepp K., Tai E.S., Goh L., Davila S., Catela Ivkovic T., Calin G.A., Voorhoeve P.M. Allele frequencies of variants in ultra conserved elements identify selective pressure on transcription factor binding. PLoS One. 2014; 9:e110692. 25369454 10.1371/journal.pone.0110692 PMC4219694

[78] Polychronopoulos D., Krithara A., Nikolaou C., Paliouras G., Almirantis Y., Giannakopoulos G. Dediu A-H, Martín-Vide C, Truthe B Analysis and classification of constrained DNA elements with n-gram graphs and genomic signatures. Algorithms for Computational Biology, Lecture Notes in Computer Science. 2014; 8542:Cham: Springer International Publishing 220–234.

[79] Polychronopoulos D., Weitschek E., Dimitrieva S., Bucher P., Felici G., Almirantis Y. Classification of selectively constrained DNA elements using feature vectors and rule-based classifiers. Genomics. 2014; 104:79–86. 25058025 10.1016/j.ygeno.2014.07.004

[80] Engelhardt B.E., Brown C.D. Diving deeper to predict noncoding sequence function. Nat. Methods. 2015; 12:925–926. 26418766 10.1038/nmeth.3604

[81] Angermueller C., Pärnamaa T., Parts L., Stegle O. Deep learning for computational biology. Mol. Syst. Biol. 2016; 12:878. 27474269 10.15252/msb.20156651 PMC4965871

[82] LeCun Y., Bengio Y., Hinton G. Deep learning. Nature. 2015; 521:436–444. 26017442 10.1038/nature14539

[83] Faircloth B.C., McCormack J.E., Crawford N.G., Harvey M.G., Brumfield R.T., Glenn T.C. Ultraconserved elements anchor thousands of genetic markers spanning multiple evolutionary timescales. Syst. Biol. 2012; 61:717–726. 22232343 10.1093/sysbio/sys004

[84] McCormack J.E., Faircloth B.C., Crawford N.G., Gowaty P.A., Brumfield R.T., Glenn T.C. Ultraconserved elements are novel phylogenomic markers that resolve placental mammal phylogeny when combined with species-tree analysis. Genome Res. 2012; 22:746–754. 22207614 10.1101/gr.125864.111 PMC3317156

[85] Starrett J., Derkarabetian S., Hedin M., Bryson R.W., McCormack J.E., Faircloth B.C. High phylogenetic utility of an ultraconserved element probe set designed for Arachnida. Mol. Ecol. Resour. 2016; 17:812–823. 27768256 10.1111/1755-0998.12621

[86] Harvey M.G., Smith B.T., Glenn T.C., Faircloth B.C., Brumfield R.T. Sequence capture versus restriction site associated DNA sequencing for shallow systematics. Syst. Biol. 2016; 65:910–924. 27288477 10.1093/sysbio/syw036

[87] McCormack J.E., Tsai W.L.E., Faircloth B.C. Sequence capture of ultraconserved elements from bird museum specimens. Mol. Ecol. Resour. 2016; 16:1189–1203. 26391430 10.1111/1755-0998.12466

[88] Faircloth B.C. PHYLUCE is a software package for the analysis of conserved genomic loci. Bioinformatics. 2016; 32:786–788. 26530724 10.1093/bioinformatics/btv646

[89] Davies K.T.J., Tsagkogeorga G., Rossiter S.J. Divergent evolutionary rates in vertebrate and mammalian specific conserved non-coding elements (CNEs) in echolocating mammals. BMC Evol. Biol. 2014; 14:261. 25523630 10.1186/s12862-014-0261-5 PMC4302572

[90] Esselstyn J.A., Oliveros C.H., Swanson M.T., Faircloth B.C. Investigating difficult nodes in the placental mammal tree with expanded taxon sampling and thousands of ultraconserved elements. Genome Biol. Evol. 2017; 9:2308–2321. 28934378 10.1093/gbe/evx168 PMC5604124

[91] Edwards S.V., Cloutier A., Baker A.J. Conserved non-exonic elements: a novel class of marker for phylogenomics. Syst. Biol. 2017; 66:1028–1044. 28637293 10.1093/sysbio/syx058 PMC5790140

[92] Benko S., Fantes J.A., Amiel J., Kleinjan D.-J., Thomas S., Ramsay J., Jamshidi N., Essafi A., Heaney S., Gordon C.T. Highly conserved non-coding elements on either side of SOX9 associated with Pierre Robin sequence. Nat. Genet. 2009; 41:359–364. 19234473 10.1038/ng.329

[93] Rahimov F., Marazita M.L., Visel A., Cooper M.E., Hitchler M.J., Rubini M., Domann F.E., Govil M., Christensen K., Bille C. Disruption of an AP-2alpha binding site in an IRF6 enhancer is associated with cleft lip. Nat. Genet. 2008; 40:1341–1347. 18836445 10.1038/ng.242 PMC2691688

[94] Roessler E., Hu P., Hong S.-K., Srivastava K., Carrington B., Sood R., Petrykowska H., Elnitski L., Ribeiro L.A., Richieri-Costa A. Unique alterations of an ultraconserved non-coding element in the 3′UTR of ZIC2 in holoprosencephaly. PLoS One. 2012; 7:e39026. 22859937 10.1371/journal.pone.0039026 PMC3409191

[95] Lettice L.A., Horikoshi T., Heaney S.J.H., van Baren M.J., van der Linde H.C., Breedveld G.J., Joosse M., Akarsu N., Oostra B.A., Endo N. Disruption of a long-range cis-acting regulator for Shh causes preaxial polydactyly. Proc. Natl. Acad. Sci. U.S.A. 2002; 99:7548–7553. 12032320 10.1073/pnas.112212199 PMC124279

[96] Emison E.S., McCallion A.S., Kashuk C.S., Bush R.T., Grice E., Lin S., Portnoy M.E., Cutler D.J., Green E.D., Chakravarti A. A common sex-dependent mutation in a RET enhancer underlies Hirschsprung disease risk. Nature. 2005; 434:857–863. 15829955 10.1038/nature03467

[97] Justice C.M., Bishop K., Carrington B., Mullikin J.C., Swindle K., Marosy B., Sood R., Miller N.H., Wilson A.F. Evaluation of IRX genes and conserved noncoding elements in a region on 5p13.3 linked to families with Familial Idiopathic Scoliosis and Kyphosis. G3 (Bethesda). 2016; 6:1707–1712. 27172222 10.1534/g3.116.029975 PMC4889666

[98] Martinez A.F., Abe Y., Hong S., Molyneux K., Yarnell D., Löhr H., Driever W., Acosta M.T., Arcos-Burgos M., Muenke M. An ultraconserved brain-specific enhancer within ADGRL3 (LPHN3) underpins attention-deficit/hyperactivity disorder susceptibility. Biol. Psychiatry. 2016; 80:943–954. 27692237 10.1016/j.biopsych.2016.06.026 PMC5108697

[99] Doan R.N., Bae B.-I., Cubelos B., Chang C., Hossain A.A., Al-Saad S., Mukaddes N.M., Oner O., Al-Saffar M., Balkhy S. Mutations in human accelerated regions disrupt cognition and social behavior. Cell. 2016; 167:341–354. 27667684 10.1016/j.cell.2016.08.071 PMC5063026

[100] Spieler D., Kaffe M., Knauf F., Bessa J., Tena J.J., Giesert F., Schormair B., Tilch E., Lee H., Horsch M. Restless legs syndrome-associated intronic common variant in Meis1 alters enhancer function in the developing telencephalon. Genome Res. 2014; 24:592–603. 24642863 10.1101/gr.166751.113 PMC3975059

[101] Dathe K., Kjaer K.W., Brehm A., Meinecke P., Nürnberg P., Neto J.C., Brunoni D., Tommerup N., Ott C.E., Klopocki E. Duplications involving a conserved regulatory element downstream of BMP2 are associated with brachydactyly type A2. Am. J. Hum. Genet. 2009; 84:483–492. 19327734 10.1016/j.ajhg.2009.03.001 PMC2667973

[102] Kurth I., Klopocki E., Stricker S., van Oosterwijk J., Vanek S., Altmann J., Santos H.G., van Harssel J.J.T., de Ravel T., Wilkie A.O.M. Duplications of noncoding elements 5′ of SOX9 are associated with brachydactyly-anonychia. Nat. Genet. 2009; 41:862–863. 19639023 10.1038/ng0809-862

[103] Klopocki E., Lohan S., Brancati F., Koll R., Brehm A., Seemann P., Dathe K., Stricker S., Hecht J., Bosse K. Copy-number variations involving the IHH locus are associated with syndactyly and craniosynostosis. Am. J. Hum. Genet. 2011; 88:70–75. 21167467 10.1016/j.ajhg.2010.11.006 PMC3014361

[104] Kleinjan D.A., Seawright A., Schedl A., Quinlan R.A., Danes S., van Heyningen V. Aniridia-associated translocations, DNase hypersensitivity, sequence comparison and transgenic analysis redefine the functional domain of PAX6. Hum. Mol. Genet. 2001; 10:2049–2059. 11590122 10.1093/hmg/10.19.2049

[105] de Kok Y.J., Vossenaar E.R., Cremers C.W., Dahl N., Laporte J., Hu L.J., Lacombe D., Fischel-Ghodsian N., Friedman R.A., Parnes L.S. Identification of a hot spot for microdeletions in patients with X-linked deafness type 3 (DFN3) 900 kb proximal to the DFN3 gene POU3F4. Hum. Mol. Genet. 1996; 5:1229–1235. 8872461 10.1093/hmg/5.9.1229

[106] Beysen D., Raes J., Leroy B.P., Lucassen A., Yates J.R.W., Clayton-Smith J., Ilyina H., Brooks S.S., Christin-Maitre S., Fellous M. Deletions involving long-range conserved nongenic sequences upstream and downstream of FOXL2 as a novel disease-causing mechanism in blepharophimosis syndrome. Am. J. Hum. Genet. 2005; 77:205–218. 15962237 10.1086/432083 PMC1224524

[107] D’haene B., Attanasio C., Beysen D., Dostie J., Lemire E., Bouchard P., Field M., Jones K., Lorenz B., Menten B. Disease-causing 7.4 kb cis-regulatory deletion disrupting conserved non-coding sequences and their interaction with the FOXL2 promotor: implications for mutation screening. PLoS Genet. 2009; 5:e1000522. 19543368 10.1371/journal.pgen.1000522 PMC2689649

[108] Bondurand N., Fouquet V., Baral V., Lecerf L., Loundon N., Goossens M., Duriez B., Labrune P., Pingault V. Alu-mediated deletion of SOX10 regulatory elements in Waardenburg syndrome type 4. Eur. J. Hum. Genet. 2012; 20:990–994. 22378281 10.1038/ejhg.2012.29 PMC3421117

[109] Amiel J., Benko S., Gordon C.T., Lyonnet S. Disruption of long-distance highly conserved noncoding elements in neurocristopathies. Ann. N Y Acad. Sci. 2010; 1214:34–46. 21175683 10.1111/j.1749-6632.2010.05878.x

[110] Nóbrega M.A., Zhu Y., Plajzer-Frick I., Afzal V., Rubin E.M. Megabase deletions of gene deserts result in viable mice. Nature. 2004; 431:988–993. 15496924 10.1038/nature03022

[111] Ahituv N., Zhu Y., Visel A., Holt A., Afzal V., Pennacchio L.A., Rubin E.M. Deletion of ultraconserved elements yields viable mice. PLoS Biol. 2007; 5:e234. 17803355 10.1371/journal.pbio.0050234 PMC1964772

[112] Pennisi E. Mysterious unchanging DNA finds a purpose in life. Science. 2017; 356:892. 28572342 10.1126/science.356.6341.892

[113] Calin G.A., Liu C., Ferracin M., Hyslop T., Spizzo R., Sevignani C., Fabbri M., Cimmino A., Lee E.J., Wojcik S.E. Ultraconserved regions encoding ncRNAs are altered in human leukemias and carcinomas. Cancer Cell. 2007; 12:215–229. 17785203 10.1016/j.ccr.2007.07.027

[114] Braconi C., Valeri N., Kogure T., Gasparini P., Huang N., Nuovo G.J., Terracciano L., Croce C.M., Patel T. Expression and functional role of a transcribed noncoding RNA with an ultraconserved element in hepatocellular carcinoma. Proc. Natl. Acad. Sci. U.S.A. 2011; 108:786–791. 21187392 10.1073/pnas.1011098108 PMC3021052

[115] Bao B.-Y., Lin V.C., Yu C.-C., Yin H.-L., Chang T.-Y., Lu T.-L., Lee H.-Z., Pao J.-B., Huang C.-Y., Huang S.-P. Genetic variants in ultraconserved regions associate with prostate cancer recurrence and survival. Sci. Rep. 2016; 6:22124. 26902966 10.1038/srep22124 PMC4763269

[116] Hudson R.S., Yi M., Volfovsky N., Prueitt R.L., Esposito D., Volinia S., Liu C.-G., Schetter A.J., Van Roosbroeck K., Stephens R.M. Transcription signatures encoded by ultraconserved genomic regions in human prostate cancer. Mol. Cancer. 2013; 12:13. 23409773 10.1186/1476-4598-12-13 PMC3626580

[117] Sana J., Hankeova S., Svoboda M., Kiss I., Vyzula R., Slaby O. Expression levels of transcribed ultraconserved regions uc.73 and uc.388 are altered in colorectal cancer. Oncology. 2012; 82:114–118. 22328099 10.1159/000336479

[118] Mestdagh P., Fredlund E., Pattyn F., Rihani A., Van Maerken T., Vermeulen J., Kumps C., Menten B., De Preter K., Schramm A. An integrative genomics screen uncovers ncRNA T-UCR functions in neuroblastoma tumours. Oncogene. 2010; 29:3583–3592. 20383195 10.1038/onc.2010.106

[119] Scaruffi P., Stigliani S., Moretti S., Coco S., De Vecchi C., Valdora F., Garaventa A., Bonassi S., Tonini G.P. Transcribed-ultra conserved region expression is associated with outcome in high-risk neuroblastoma. BMC Cancer. 2009; 9:441. 20003513 10.1186/1471-2407-9-441 PMC2804711

[120] Fassan M., Dall’Olmo L., Galasso M., Braconi C., Pizzi M., Realdon S., Volinia S., Valeri N., Gasparini P., Baffa R. Transcribed ultraconserved noncoding RNAs (T-UCR) are involved in Barrett's esophagus carcinogenesis. Oncotarget. 2014; 5:7162–7171. 25216530 10.18632/oncotarget.2249 PMC4196192

[121] Olivieri M., Ferro M., Terreri S., Durso M., Romanelli A., Avitabile C., De Cobelli O., Messere A., Bruzzese D., Vannini I. Long non-coding RNA containing ultraconserved genomic region 8 promotes bladder cancer tumorigenesis. Oncotarget. 2016; 7:20636–20654. 26943042 10.18632/oncotarget.7833 PMC4991481

[122] Qian X.X., Peng J.C., Xu A.T., Zhao D., Qiao Y.Q., Wang T.R., Shen J., Ran Z.H. Noncoding transcribed ultraconserved region (T-UCR) uc.261 participates in intestinal mucosa barrier damage in crohn's disease. Inflamm. Bowel Dis. 2016; 22:2840–2852. 27846191 10.1097/MIB.0000000000000945

[123] Ragvin A., Moro E., Fredman D., Navratilova P., Drivenes Ø., Engström P.G., Alonso M.E., de la Calle Mustienes E., Gómez Skarmeta J.L., Tavares M.J. Long-range gene regulation links genomic type 2 diabetes and obesity risk regions to HHEX, SOX4, and IRX3. Proc. Natl. Acad. Sci. U.S.A. 2010; 107:775–780. 20080751 10.1073/pnas.0911591107 PMC2818943

[124] Smemo S., Tena J.J., Kim K.-H., Gamazon E.R., Sakabe N.J., Gómez-Marín C., Aneas I., Credidio F.L., Sobreira D.R., Wasserman N.F. Obesity-associated variants within FTO form long-range functional connections with IRX3. Nature. 2014; 507:371–375. 24646999 10.1038/nature13138 PMC4113484

[125] Claussnitzer M., Dankel S.N., Kim K.H., Quon G., Meuleman W., Haugen C., Glunk V., Sousa I.S., Beaudry J.L., Puviindran V. FTO obesity variant circuitry and adipocyte browning in humans. N Engl. J. Med. 2015; 373:895–907. 26287746 10.1056/NEJMoa1502214 PMC4959911

[126] Navratilova P., Becker T.S. Genomic regulatory blocks in vertebrates and implications in human disease. Brief. Funct. Genomic Proteomic. 2009; 8:333–342. 19561171 10.1093/bfgp/elp019

[127] Zhang F., Lupski J.R. Non-coding genetic variants in human disease. Hum. Mol. Genet. 2015; 24:R102–R110. 26152199 10.1093/hmg/ddv259 PMC4572001

[128] Maurano M.T., Humbert R., Rynes E., Thurman R.E., Haugen E., Wang H., Reynolds A.P., Sandstrom R., Qu H., Brody J. Systematic localization of common disease-associated variation in regulatory DNA. Science. 2012; 337:1190–1195. 22955828 10.1126/science.1222794 PMC3771521

[129] Harmston N., Baresic A., Lenhard B. The mystery of extreme non-coding conservation. Philos. Trans. R. Soc. Lond. B Biol. Sci. 2013; 368:20130021. 24218634 10.1098/rstb.2013.0021 PMC3826495

[130] Drake J.A., Bird C., Nemesh J., Thomas D.J., Newton-Cheh C., Reymond A., Excoffier L., Attar H., Antonarakis S.E., Dermitzakis E.T. Conserved noncoding sequences are selectively constrained and not mutation cold spots. Nat. Genet. 2006; 38:223–227. 16380714 10.1038/ng1710

[131] Katzman S., Kern A.D., Bejerano G., Fewell G., Fulton L., Wilson R.K., Salama S.R., Haussler D. Human genome ultraconserved elements are ultraselected. Science. 2007; 317:915. 17702936 10.1126/science.1142430

[132] Casillas S., Barbadilla A., Bergman C.M. Purifying selection maintains highly conserved noncoding sequences in Drosophila. Mol. Biol. Evol. 2007; 24:2222–2234. 17646256 10.1093/molbev/msm150

[133] De Silva D.R., Nichols R., Elgar G. Purifying selection in deeply conserved human enhancers is more consistent than in coding sequences. PLoS One. 2014; 9:e103357. 25062004 10.1371/journal.pone.0103357 PMC4111549

[134] Bejerano G., Lowe C.B., Ahituv N., King B., Siepel A., Salama S.R., Rubin E.M., Kent W.J., Haussler D. A distal enhancer and an ultraconserved exon are derived from a novel retroposon. Nature. 2006; 441:87–90. 16625209 10.1038/nature04696

[135] Lowe C.B., Bejerano G., Haussler D. Thousands of human mobile element fragments undergo strong purifying selection near developmental genes. Proc. Natl. Acad. Sci. U.S.A. 2007; 104:8005–8010. 17463089 10.1073/pnas.0611223104 PMC1876562

[136] Kamal M., Xie X., Lander E.S. A large family of ancient repeat elements in the human genome is under strong selection. Proc. Natl. Acad. Sci. U.S.A. 2006; 103:2740–2745. 16477033 10.1073/pnas.0511238103 PMC1413850

[137] Dong X., Navratilova P., Fredman D., Drivenes Ø., Becker T.S., Lenhard B. Exonic remnants of whole-genome duplication reveal cis-regulatory function of coding exons. Nucleic Acids Res. 2010; 38:1071–1085. 19969543 10.1093/nar/gkp1124 PMC2831330

[138] Lampe X., Samad O.A., Guiguen A., Matis C., Remacle S., Picard J.J., Rijli F.M., Rezsohazy R. An ultraconserved Hox-Pbx responsive element resides in the coding sequence of Hoxa2 and is active in rhombomere 4. Nucleic Acids Res. 2008; 36:3214–3225. 18417536 10.1093/nar/gkn148 PMC2425489

[139] Mikkelsen T.S., Wakefield M.J., Aken B., Amemiya C.T., Chang J.L., Duke S., Garber M., Gentles A.J., Goodstadt L., Heger A. Genome of the marsupial Monodelphis domestica reveals innovation in non-coding sequences. Nature. 2007; 447:167–177. 17495919 10.1038/nature05805

[140] Babarinde I.A., Saitou N. Heterogeneous tempo and mode of conserved noncoding sequence evolution among four mammalian orders. Genome Biol. Evol. 2013; 5:2330–2343. 24259317 10.1093/gbe/evt177 PMC3879966

[141] Takahashi M., Saitou N. Identification and characterization of lineage-specific highly conserved noncoding sequences in mammalian genomes. Genome Biol. Evol. 2012; 4:641–657. 22505575 10.1093/gbe/evs035 PMC3381673

[142] Lowe C.B., Kellis M., Siepel A., Raney B.J., Clamp M., Salama S.R., Kingsley D.M., Lindblad-Toh K., Haussler D. Three periods of regulatory innovation during vertebrate evolution. Science. 2011; 333:1019–1024. 21852499 10.1126/science.1202702 PMC3511857

[143] McEwen G.K., Goode D.K., Parker H.J., Woolfe A., Callaway H., Elgar G. Early evolution of conserved regulatory sequences associated with development in vertebrates. PLoS Genet. 2009; 5:e1000762. 20011110 10.1371/journal.pgen.1000762 PMC2781166

[144] Parker H.J., Piccinelli P., Sauka-Spengler T., Bronner M., Elgar G. Ancient Pbx-Hox signatures define hundreds of vertebrate developmental enhancers. BMC Genomics. 2011; 12:637. 22208168 10.1186/1471-2164-12-637 PMC3261376

[145] McLean C.Y., Reno P.L., Pollen A.A., Bassan A.I., Capellini T.D., Guenther C., Indjeian V.B., Lim X., Menke D.B., Schaar B.T. Human-specific loss of regulatory DNA and the evolution of human-specific traits. Nature. 2011; 471:216–219. 21390129 10.1038/nature09774 PMC3071156

[146] Indjeian V.B., Kingman G.A., Jones F.C., Guenther C.A., Grimwood J., Schmutz J., Myers R.M., Kingsley D.M. Evolving new skeletal traits by cis-regulatory changes in bone morphogenetic proteins. Cell. 2016; 164:45–56. 26774823 10.1016/j.cell.2015.12.007 PMC4759241

[147] Infante C.R., Mihala A.G., Park S., Wang J.S., Johnson K.K., Lauderdale J.D., Menke D.B. Shared enhancer activity in the limbs and phallus and functional divergence of a limb-genital cis-regulatory element in snakes. Dev. Cell. 2015; 35:107–119. 26439399 10.1016/j.devcel.2015.09.003 PMC4605891

[148] Kvon E.Z., Kamneva O.K., Melo U.S., Barozzi I., Osterwalder M., Mannion B.J., Tissières V., Pickle C.S., Plajzer-Frick I., Lee E.A. Progressive loss of function in a limb enhancer during snake evolution. Cell. 2016; 167:633–642. 27768887 10.1016/j.cell.2016.09.028 PMC5484524

[149] Leal F., Cohn M.J. Loss and re-emergence of legs in snakes by modular evolution of Sonic hedgehog and HOXD enhancers. Curr. Biol. 2016; 26:2966–2973. 27773569 10.1016/j.cub.2016.09.020

[150] Sagai T., Masuya H., Tamura M., Shimizu K., Yada Y., Wakana S., Gondo Y., Noda T., Shiroishi T. Phylogenetic conservation of a limb-specific, cis-acting regulator of Sonic hedgehog (Shh). Mamm. Genome. 2004; 15:23–34. 14727139 10.1007/s00335-033-2317-5

[151] Chan Y.F., Marks M.E., Jones F.C., Villarreal G., Shapiro M.D., Brady S.D., Southwick A.M., Absher D.M., Grimwood J., Schmutz J. Adaptive evolution of pelvic reduction in sticklebacks by recurrent deletion of a Pitx1 enhancer. Science. 2010; 327:302–305. 20007865 10.1126/science.1182213 PMC3109066

[152] Hiller M., Schaar B.T., Bejerano G. Hundreds of conserved non-coding genomic regions are independently lost in mammals. Nucleic Acids Res. 2012; 40:11463–11476. 23042682 10.1093/nar/gks905 PMC3526296

[153] Marcovitz A., Jia R., Bejerano G. “reverse genomics” predicts function of human conserved noncoding elements. Mol. Biol. Evol. 2016; 33:1358–1369. 26744417 10.1093/molbev/msw001 PMC4909134

[154] Lee A.P., Kerk S.Y., Tan Y.Y., Brenner S., Venkatesh B. Ancient vertebrate conserved noncoding elements have been evolving rapidly in teleost fishes. Mol. Biol. Evol. 2011; 28:1205–1215. 21081479 10.1093/molbev/msq304

[155] Lin Q., Fan S., Zhang Y., Xu M., Zhang H., Yang Y., Lee A.P., Woltering J.M., Ravi V., Gunter H.M. The seahorse genome and the evolution of its specialized morphology. Nature. 2016; 540:395–399. 27974754 10.1038/nature20595 PMC8127814

[156] Franchini L.F., López-Leal R., Nasif S., Beati P., Gelman D.M., Low M.J., de Souza F.J.S., Rubinstein M. Convergent evolution of two mammalian neuronal enhancers by sequential exaptation of unrelated retroposons. Proc. Natl. Acad. Sci. U.S.A. 2011; 108:15270–15275. 21876128 10.1073/pnas.1104997108 PMC3174587

[157] Domené S., Bumaschny V.F., de Souza F.S.J., Franchini L.F., Nasif S., Low M.J., Rubinstein M. Enhancer turnover and conserved regulatory function in vertebrate evolution. Philos. Trans. R. Soc. Lond. B Biol. Sci. 2013; 368:20130027. 24218639 10.1098/rstb.2013.0027 PMC3826500

[158] Weirauch M.T., Hughes T.R. Conserved expression without conserved regulatory sequence: the more things change, the more they stay the same. Trends Genet. 2010; 26:66–74. 20083321 10.1016/j.tig.2009.12.002

[159] Gittelman R.M., Hun E., Ay F., Madeoy J., Pennacchio L., Noble W.S., Hawkins R.D., Akey J.M. Comprehensive identification and analysis of human accelerated regulatory DNA. Genome Res. 2015; 25:1245–1255. 26104583 10.1101/gr.192591.115 PMC4561485

[160] Pollard K.S., Salama S.R., Lambert N., Lambot M.-A., Coppens S., Pedersen J.S., Katzman S., King B., Onodera C., Siepel A. An RNA gene expressed during cortical development evolved rapidly in humans. Nature. 2006; 443:167–172. 16915236 10.1038/nature05113

[161] Dong X., Wang X., Zhang F., Tian W. Genome-wide identification of regulatory sequences undergoing accelerated evolution in the human genome. Mol. Biol. Evol. 2016; 33:2565–2575. 27401230 10.1093/molbev/msw128 PMC5026254

[162] Bird C.P., Stranger B.E., Liu M., Thomas D.J., Ingle C.E., Beazley C., Miller W., Hurles M.E., Dermitzakis E.T. Fast-evolving noncoding sequences in the human genome. Genome Biol. 2007; 8:R118. 17578567 10.1186/gb-2007-8-6-r118 PMC2394770

[163] Prabhakar S., Noonan J.P., Pääbo S., Rubin E.M. Accelerated evolution of conserved noncoding sequences in humans. Science. 2006; 314:786. 17082449 10.1126/science.1130738

[164] Hubisz M.J., Pollard K.S. Exploring the genesis and functions of human accelerated regions sheds light on their role in human evolution. Curr. Opin. Genet. Dev. 2014; 29:15–21. 25156517 10.1016/j.gde.2014.07.005

[165] Boyd J.L., Skove S.L., Rouanet J.P., Pilaz L.-J., Bepler T., Gordân R., Wray G.A., Silver D.L. Human-chimpanzee differences in a FZD8 enhancer alter cell-cycle dynamics in the developing neocortex. Curr. Biol. 2015; 25:772–779. 25702574 10.1016/j.cub.2015.01.041 PMC4366288

[166] Prabhakar S., Visel A., Akiyama J.A., Shoukry M., Lewis K.D., Holt A., Plajzer-Frick I., Morrison H., Fitzpatrick D.R., Afzal V. Human-specific gain of function in a developmental enhancer. Science. 2008; 321:1346–1350. 18772437 10.1126/science.1159974 PMC2658639

[167] Diao Y., Li B., Meng Z., Jung I., Lee A.Y., Dixon J., Maliskova L., Guan K.-L., Shen Y., Ren B. A new class of temporarily phenotypic enhancers identified by CRISPR/Cas9-mediated genetic screening. Genome Res. 2016; 26:397–405. 26813977 10.1101/gr.197152.115 PMC4772021

[168] Klann T.S., Black J.B., Chellappan M., Safi A., Song L., Hilton I.B., Crawford G.E., Reddy T.E., Gersbach C.A. CRISPR-Cas9 epigenome editing enables high-throughput screening for functional regulatory elements in the human genome. Nat. Biotechnol. 2017; 35:561–568. 28369033 10.1038/nbt.3853 PMC5462860

[169] McEwen G.K., Woolfe A., Goode D., Vavouri T., Callaway H., Elgar G. Ancient duplicated conserved noncoding elements in vertebrates: a genomic and functional analysis. Genome Res. 2006; 16:451–465. 16533910 10.1101/gr.4143406 PMC1457030

[170] Lupiáñez D.G., Kraft K., Heinrich V., Krawitz P., Brancati F., Klopocki E., Horn D., Kayserili H., Opitz J.M., Laxova R. Disruptions of topological chromatin domains cause pathogenic rewiring of gene-enhancer interactions. Cell. 2015; 161:1012–1025. 25959774 10.1016/j.cell.2015.04.004 PMC4791538

[171] Flavahan W.A., Drier Y., Liau B.B., Gillespie S.M., Venteicher A.S., Stemmer-Rachamimov A.O., Suvà M.L., Bernstein B.E. Insulator dysfunction and oncogene activation in IDH mutant gliomas. Nature. 2016; 529:110–114. 26700815 10.1038/nature16490 PMC4831574

[172] Ibn-Salem J., Köhler S., Love M.I., Chung H.-R., Huang N., Hurles M.E., Haendel M., Washington N.L., Smedley D., Mungall C.J. Deletions of chromosomal regulatory boundaries are associated with congenital disease. Genome Biol. 2014; 15:423. 25315429 10.1186/s13059-014-0423-1 PMC4180961

[173] Valton A.-L., Dekker J. TAD disruption as oncogenic driver. Curr. Opin. Genet. Dev. 2016; 36:34–40. 27111891 10.1016/j.gde.2016.03.008 PMC4880504

