# Supplementary material for: Conserved non-coding elements: developmental gene regulation meets genome organization
Source: Nucleic Acids Res. 2017 Nov 7;45(22):12611–24. doi: 10.1093/nar/gkx1074 (PMC5728398; doi:10.1093/nar/gkx1074)
Supplement: Supplementary Data [file gkx1074_supp.pdf]

Supplementary Table 1: Nomenclature for various sets of conserved non-coding elements

Abbreviation	Description	Identification	Reference
ANCOR	Ancestral non-coding conserved region	various computational methods	(1)
CNC	Conserved non-coding	≥ 70% seq. id. over 100 nucleotides between human and mouse; coding regions are removed	(2)
CNE	Conserved non-coding elements	local sequence similarity between human and *Fugu* for over 100 nucleotides; coding and repetitive regions are removed	(3)
CNEE	Conserved non-exonic elements	phastCons (a phylogenetic hidden markov model) was applied on multiple alignments of 40 vertebrate genomes	(4)
CNG	Conserved non-genic	≥ 70% seq. id. over 100 nucleotides between human and mouse; strictly non-coding	(5)
CNS	Conserved non-coding sequences	various computational methods	(6)
EC	Extremely conserved elements	≥ 80% seq. id. over 100 nucleotides in 40 vertebrate genomes	(7)
HCE	Highly Conserved Elements	union set of sequences identified in other studies: UCEs (8), UCR (9) and CNE (3)	(10)
HCR	Highly conserved regions	≥ 70% seq. id. over 100 nucleotides between classes of vertebrates	(11)
HCNE	Highly conserved non-coding elements	sequence stretches over 50 nucleotides, where the probability of being under negative selection given the conservation score in human and dog comparison, is over 95%	(12)
HCNR	Highly conserved non-coding regions	≥ 70% seq. id. over 100 nucleotides in human, mouse, chicken, Xenopus and *Fugu* genomes; coding regions are removed	(13)
LCNS	Long conserved non-coding sequences	long sequences (≥ 500 nucleotides) showing ≥ 95% similarity between human and mouse; coding regions are removed	(14)
LIME	Long identical multispecies elements	extremely conserved elements over 100 nucleotides for six animal and six large plant genomes	(15)

MCS	Multispecies conserved sequences	conservation score threshold was set so that the detected sequences contain 5% of the human genome (rough estimate of the mammalian genome under selective pressure), minimum length being 25 nucleotides	(16)
PCNE	Phylogenetically Conserved non-coding elements	sequences with local sequence similarity for over 45 nucleotides between vertebrates and amphioxus; coding and repetitive regions are removed	(17)
UCE	Ultraconserved elements	sequences having 100% similarity over 200 nucleotides between human, mouse and rat genomes; 25% of those overlap known protein-coding sequences	(8)
UCR	Ultraconserved regions	≥ 95% similarity over 50 nucleotides between human and mouse genomes, also partially aligned to fugu; coding regions are removed	(9)

References

1. Aloni,R. and Lancet,D. (2005) Conservation anchors in the vertebrate genome. *Genome Biol*, **6**, 115.

2. Couronne,O., Poliakov,A., Bray,N., Ishkhanov,T., Ryaboy,D., Rubin,E., Pachter,L. and Dubchak,I. (2003) Strategies and tools for whole-genome alignments. *Genome Res*, **13**, 73–80.

3. Woolfe,A., Goodson,M., Goode,D.K., Snell,P., McEwen,G.K., Vavouri,T., Smith,S.F., North,P., Callaway,H., Kelly,K., *et al.* (2005) Highly conserved non-coding sequences are associated with vertebrate development. *PLoS Biol*, **3**, e7.

4. Lowe,C.B., Kellis,M., Siepel,A., Raney,B.J., Clamp,M., Salama,S.R., Kingsley,D.M., Lindblad-Toh,K. and Haussler,D. (2011) Three periods of regulatory innovation during vertebrate evolution. *Science*, **333**, 1019–1024.

5. Dermitzakis,E.T., Reymond,A., Lyle,R., Scamuffa,N., Ucla,C., Deutsch,S., Stevenson,B.J., Flegel,V., Bucher,P., Jongeneel,C.V., *et al.* (2002) Numerous potentially functional but non-genic conserved sequences on human chromosome 21. *Nature*, **420**, 578–582.

6. Dubchak,I., Brudno,M., Loots,G.G., Pachter,L., Mayor,C., Rubin,E.M. and Frazer,K.A. (2000) Active conservation of noncoding sequences revealed by three-way species comparisons. *Genome Res*, **10**, 1304–1306.

7. Tseng,H.-H.E. and Tompa,M. (2009) Algorithms for locating extremely conserved elements in multiple sequence alignments. *BMC Bioinformatics*, **10**, 432.

8. Bejerano,G., Pheasant,M., Makunin,I., Stephen,S., Kent,W.J., Mattick,J.S. and Haussler,D. (2004)

Ultraconserved elements in the human genome. *Science*, **304**, 1321–1325.

9. Sandelin,A., Bailey,P., Bruce,S., Engström,P.G., Klos,J.M., Wasserman,W.W., Ericson,J. and Lenhard,B. (2004) Arrays of ultraconserved non-coding regions span the loci of key developmental genes in vertebrate genomes. *BMC Genomics*, **5**, 99.

10. Sun,H., Skogerbø,G. and Chen,R. (2006) Conserved distances between vertebrate highly conserved elements. *Hum Mol Genet*, **15**, 2911–2922.

11. Duret,L., Dorkeld,F. and Gautier,C. (1993) Strong conservation of non-coding sequences during vertebrates evolution: potential involvement in post-transcriptional regulation of gene expression. *Nucleic Acids Res*, **21**, 2315–2322.

12. Lindblad-Toh,K., Wade,C.M., Mikkelsen,T.S., Karlsson,E.K., Jaffe,D.B., Kamal,M., Clamp,M., Chang,J.L., Kulbokas,E.J., Zody,M.C., *et al.* (2005) Genome sequence, comparative analysis and haplotype structure of the domestic dog. *Nature*, **438**, 803–819.

13. de la Calle-Mustienes,E., Feijóo,C.G., Manzanares,M., Tena,J.J., Rodríguez-Seguel,E., Letizia,A., Allende,M.L. and Gómez-Skarmeta,J.L. (2005) A functional survey of the enhancer activity of conserved non-coding sequences from vertebrate Iroquois cluster gene deserts. *Genome Res*, **15**, 1061–1072.

14. Sakuraba,Y., Kimura,T., Masuya,H., Noguchi,H., Sezutsu,H., Takahasi,K.R., Toyoda,A., Fukumura,R., Murata,T., Sakaki,Y., *et al.* (2008) Identification and characterization of new long conserved noncoding sequences in vertebrates. *Mamm Genome*, **19**, 703–712.

15. Reneker,J., Lyons,E., Conant,G.C., Pires,J.C., Freeling,M., Shyu,C.-R. and Korkin,D. (2012) Long identical multispecies elements in plant and animal genomes. *Proc Natl Acad Sci U S A*, **109**, E1183-91.

16. Margulies,E.H., Blanchette,M., NISC Comparative Sequencing Program, Haussler,D. and Green,E.D. (2003) Identification and characterization of multi-species conserved sequences. *Genome Res*, **13**, 2507–2518.

17. Hufton,A.L., Mathia,S., Braun,H., Georgi,U., Lehrach,H., Vingron,M., Poustka,A.J. and Panopoulou,G. (2009) Deeply conserved chordate noncoding sequences preserve genome synteny but do not drive gene duplicate retention. *Genome Res*, **19**, 2036–2051.
